# Comparative Study of Two Insulinlike Proteases in *Cryptosporidium parvum*

**DOI:** 10.3390/microorganisms9040861

**Published:** 2021-04-16

**Authors:** Wei He, Cong Lai, Fuxian Yang, Yu Li, Na Li, Yaqiong Guo, Ziding Zhang, Lihua Xiao, Yaoyu Feng

**Affiliations:** 1 Center for Emerging and Zoonotic Diseases, College of Veterinary Medicine, South China Agricultural University, Guangzhou, Guangdong 510642, China; hwhewei0707@163.com (W.H.); FuxianYang1210@163.com (F.Y.); nli@scau.edu.cn (N.L.); guoyaqiong1987@sina.com (Y.G.); 2 State Key Laboratory of Bioreactor Engineering, School of Resources and Environmental Engineering, East China University of Science and Technology, Shanghai 200237, China; cong_lai0905@outlook.com; 3 State Key Laboratory of Agrobiotechnology, College of Biological Sciences, China Agricultural University, Beijing 100083, China; yu_li_protein@cau.edu.cn (Y.L.); zidingzhang@cau.edu.cn (Z.Z.); 4 Guangdong Laboratory for Lingnan Modern Agriculture, Guangzhou 510642, China

**Keywords:** *Cryptosporidium parvum*, insulinlike proteases, INS-4, INS-6, invasion

## Abstract

*Cryptosporidiumparvum* is a common protozoan pathogen responsible for moderate-to-severe diarrhea in humans and animals. The small genome of *C. parvum* has 22 genes encoding insulinlike proteases (INS) with diverse sequences, suggesting that members of the protein family may have different biological functions in the life cycle. In this study, two members of the INS family, CpINS-4 and CpINS-6 with the Zn^2+^-binding motif “HXXEH” but different numbers of function domains, were expressed in *Escherichia coli* and used in the generation of polyclonal antibodies. In both recombinant and native proteins, CpINS-4 and CpINS-6 were spliced into multiple fragments. The antibodies generated recognized their respective recombinant and native proteins and the spliced products, but had minimum cross-reactivity with each other. Anti-CpINS-4 antibodies reacted with the middle region of sporozoites and merozoites, while CpINS-6 had the highest reactivity to the apical region. Polyclonal anti-CpINS-4 antibodies produced 36% reduction in parasite load in HCT-8 cultures at 24 h, while those against CpINS-6, which has one of the function domains missing, failed in doing so. The genes encoding both CpINS-4 and CpINS-6 had the highest expression in the invasion phase of in vitro *C. parvum* culture. These data suggest that CpINS-4 and CpINS-6 might be expressed in different organelles and play different biological functions in the life cycle of *C. parvum*.

## 1. Introduction

*Cryptosporidium* spp. are intracellular protozoan parasites that cause moderate-to-severe diarrhea in humans and various animals. Two *Cryptosporidium* species, *C. parvum* and *C. hominis*, are the major causative agents of human cryptosporidiosis [1]. Children, neonatal animals, and immunocompromised individuals are especially susceptible to infection [2]. In low-income countries, cryptosporidiosis is a common cause of diarrhea-associated mortality in young children [3]. It was estimated that in 2016 alone, acute *Cryptosporidium* infections caused more than 48,000 deaths and over 4.2 million disability-adjusted life-years in low- and middle-income countries [4]. No effective vaccines are in clinical use, and nitazoxanide, the sole drug approved by the U.S. Food and Drug Administration for treating cryptosporidiosis, has poor efficacy in immunocompromised individuals [5].

Various proteases and protein kinases secreted by several unique organelles are thought to be involved in host cell adhesion and invasion by apicomplexans parasites [6]. Among them, insulinlike proteases (INS), members of the M16 family of metalloproteases, are characterized by the presence of a Zn^2+^-binding motif His-Xaa-Xaa-Glu-His (HXXEH) [7,8]. The M16 family of proteases includes three subfamilies, M16A, M16B and M16C. M16A and M16C proteases have large molecular masses (>110 kDa) that can be subdivided into N- and C-terminal domains [9], while M16B proteases are usually encoded by separate genes and form heterodimers after translation [10]. 

The functions of INS in apicomplexans have attracted the attention of researchers. Falcilysin in *Plasmodium falciparum*, a M16C protease, has been identified as a key component of the catabolic process, playing an important role in hemoglobin degradation [11]. Toxolysin 4 (TLN4), a micronemal M16A in *Toxoplasma gondii*, plays a potential role in host cell invasion in response to elevated calcium levels, as TLN4-knockout tachyzoites grow poorly [12]. Another INS in *T. gondii*, Toxolysin-1 (TLN1), a M16A protease secreted by the rhoptry, has been shown to participate in the initial interaction between the parasite and the host by means of protein C-terminal and prodomain cleavage, trafficking the C-terminal portion of the protein to the rhoptry for the formation of a detergent resistant complex [13].

The results of comparative genomics analysis indicate that *C. parvum* has 22 potential INS genes, 13 of which have the highest expression in early host cell infection [14,15]. In previous studies, INS-5, INS-15 and INS-20-19 were shown to be potentially involved in the early infection of *C. parvum* [16,17,18].

In this study, CpINS-4 and CpINS-6, two M16A proteases encoded by the *cgd2_930* and *cgd2_4270* genes, respectively, in chromosome 2 of *C. parvum* were studied. They were chosen because CpINS-4 is one of the few INS in *C. parvum* that have all four domains in functional M16 proteases, while CpINS-6 has one of the domains missing. Little is known of the impact of the absence of a domain on the function of INS. Therefore, we compared the biological functions of CpINS-4 and CpINS-6 in *C. parvum* infection.

## 2. Materials and Methods

### 2.1. Parasite, Host Cells and Cell Culture

Oocysts of the *C. parvum* IOWA isolate were purchased from Waterborne, Inc. (New Orleans, LA, USA). Prior to use, they were treated on ice with 0.5% sodium hypochlorite for 10 min, washed three times with PBS and excysted at 37 °C for 30 min in the presence of 0.25% trypsin and 0.75% taurocholic acid as described [19]. The sporozoites generated were collected by centrifugation at 5000× *g* and 4 °C for 10 min, washed three times with PBS at 5,000× *g* and 4 °C for 3 min, and resuspended in RPMI 1640 medium for in vitro infection.

Human ileocecal adenocarcinoma (HCT-8) cells were purchased from the Chinese Academy of Sciences, seeded into 12-well plates (5 × 10^5^), and cultured to ~80% confluence. The culture was inoculated with 5 × 10^5^ sporozoites of *C. parvum* in RPMI 1640 containing 10% fetal bovine serum (FBS) and 50 U/mL penicillin G and 50 U/mL streptomycin. 

### 2.2. Identification and Analysis of cgd2_930 and cgd2_4270 

The full-length sequences of *cgd2_930* and *cgd2_4270* genes were obtained from CryptoDB database (https://cryptodb.org/cryptodb/app, accessed on 20 December 2020). Signal peptide and transmembrane domains (TMHMM) in them were predicted using SignalP-5.0 server (http://www.cbs.dtu.dk/services/SignalP/, accessed on 20 December 2020) and TMHMM Server v. 2.0 (http://www.cbs.dtu.dk/services/TMHMM/, accessed on 20 December 2020), respectively. Pfam 32.0 (http://pfam.xfam.org/, accessed on 20 December 2020) was used to identify possible functional domains present in them. The tertiary structures of CpINS-4 and CpINS-6 were predicted using Protein Homology/analogY Recognition Engine V 2.0 (Phyre^2^) (http://www.sbg.bio.ic.ac.uk/phyre2/html/, accessed on 20 December 2020). The crystal structures of hIDE (PDB ID: 2jbu_B) and hIDE with copurified peptides [20] were selected as the template for the homology modeling.

The *cgd2_930* gene (amplicon = 3042 bp) was amplified from genomic DNA of the *C. parvum* IOWA isolate using primers 5′-CGGGATCCATGACAGAAATAA-3′ (the BamH1 restriction site underlined) and 5′-CCGCTCGAGTATAGTTATGTTAAG-3′ (the Xhol restriction site underlined), while the coding sequence of the *cgd2_4270* gene without the putative signal peptide (amplicon = 3672 bp) was amplified using primers 5′-GCGTCGACAGTATTTGGGACCGAATTC-3′ (the Sal1 restriction site underlined) and 5′-TTGCGGCCGCAAGCTTGCTATGATCGCAGGA-3′ (the Not1 restriction site underlined). The PCR was performed in a T100^TM^ Thermal Cycler (Bio-Rad, California, USA) under the following cycling conditions: 98 °C for 30 s; 30 cycles of 98 °C for 10 s, 52 °C 10 s, and 72 °C 2 min; and 72 °C for 5 min. The PCR products were inserted into the pET28a vector (Novagen, Madison, WI, USA), which was transformed into *Escherichia coli* DH5α cells. Positive colonies were identified by PCR, with the accuracy of cloned genes being verified using DNA sequence analysis.

### 2.3. Expression and Purification of CpINS-4 and CpINS-6

The recombinant plasmid containing the correct sequence of the *cgd2_930* or *cgd2_4270* gene was transformed into BL21-Star (DE3) pLysS Competent cells (Weidi Biotechnology, Shanghai, China), which were cultured in LB medium containing 100 mg/mL kanamycin. The expression of recombinant proteins was induced at 25 °C for 12 h by adding 0.5 mM isopropyl-β-D-thiogalactoside (IPTG).

For the purification of the recombinant protein, the cultured *E. coli* cells were harvested by centrifugation and resuspended in buffer A (50 mM sodium phosphate, 300 mM NaCI, pH7.4). The cell suspension was frozen-and-thawed three times and lysed by sonication at 4 °C. The lysate was centrifuged and the pellet containing the recombinant protein was dissolved in buffer B (50 mM sodium phosphate, 8 M urea, 40 mM imidazole, 300 mM NaCI, pH7.4) supplemented with a protease inhibitor cocktail (Sigma-Aldrich, St. Louis, MS, USA). The supernatant was collected by centrifugation and filtered through a 0.45-µm filter, and loaded onto histidine-tagged protein purification resins (Ni Sepharose 6 Fast Flow, GE Healthcare, Madison, WI, USA) at 4 °C and 70 rpm for 1 h. The resins were washed with 10 volumes buffer B and eluted with buffer C (50 mM sodium phosphate, 8 M urea, 200 mM imidazole, 300 mM NaCI, and pH7.4). The urea was removed by dialyzed against buffer A, and concentrated by using Amicon^®^ Ultra-15 30 K Centrifugal Filter Devices (Millipore, Billerica, MA, USA). The purity of the recombinant protein was evaluated using SDS-PAGE. The INS identity of the recombinant proteins was verified using MALDI-TOF-MS analysis (Sangon Biotech, Shanghai, China).

### 2.4. Preparation of Polyclonal Antibodies against CpINS-4 and CpINS-6

Polyclonal antibodies against CpINS-4 and CpINS-6 were raised in specific pathogen-free rabbits as described previously [16]. Briefly, rabbits were immunized subcutaneously with 250 μg of the recombinant protein. Preimmune sera prior to the primary immunization were collected as negative controls. Boost immunizations were conducted twice at 2-week intervals using 250 μg purified protein. Postimmune sera were collected from the immunized animals seven days after the last immunization. Immunoglobulin G (IgG) was isolated from pooled immune sera using protein G affinity chromatography (GE Healthcare, Madison, WI, USA). The titer and specificity of polyclonal antibodies were evaluated using ELISA and Western blot, respectively.

### 2.5. Assessment of Cross-Reactivity of Anti-CpINS-4 and Anti-CpINS-6 Antibodies

To assess the cross-reactivity of anti-CpINS-4 and anti-CpINS-6 antibodies, recombinant CpINS-4 and CpINS-6 were separated by SDS-PAGE and transferred onto PVDF membranes. After blocking with 5% nonfat milk-PBST at 4 °C overnight, the membranes were incubated with anti-CpINS-4 or anti-CpINS-6 antibodies (1:1000) for 1 h, with goat-anti-rabbit IgG Antibody (Sigma-Aldrich) (1:5000) being used as the secondary antibody. The membranes were washed three times with PBST, treated with High-sig ECL Western Blotting Substrate (Tanon, Shanghai, China), and analyzed with a Tanon 5200 chemiluminescence imaging system.

### 2.6. Western Blot Analysis of Native CpINS-4 and CpINS-6

To identify native CpINS-4 and CpINS-6, hypochlorite-treated oocysts (1 × 10^7^) were excysted as described above. The sporozoites were collected and resuspended in the RIPA Lysis and Extraction Buffer (ThermoFisher, Waltham, MA, USA) with Protease Inhibitor Cocktail (Sigma-Aldrich). After incubation at 4 °C overnight, the supernatant was collected, mixed with protein-loading buffer, and boiled for 10 min. The lysates from sporozoites were separated by SDS-PAGE and transferred onto a PVDF membrane. The membrane was processed further with antiserum (1:1000) and preimmune serum (1:1000) as described above.

### 2.7. Assessment of cgd2_930 and cgd2_4270 Gene Expression in C. parvum

HCT-8 monolayers were inoculated with hypochlorite-treated oocysts and RNA was isolated from the infected HCT-8 cultures at 2–72 h using the RNeasy Mini Kit (QIAGEN, Germantown, Maryland, USA). Afterwards, cDNA was synthesized from the RNA using the GoScript Reverse Transcription System (Promega, Beijing, China). The expression of the *cgd2_930* or *cgd2_4270* gene was assessed using qPCR, with data from the 18S rRNA gene being used in data normalization. The qPCR was performed in a 20-μL reaction containing 0.1 mM primers, 1 µL of cDNA and 10 µL of SYBR Green PCR Mix (TOYOBO, Osaka, Japan) in a Light Cycler 480 (Roche, Basel, Switzerland), with the following cycling condition: 95 °C for 3 min and 45 cycles of 95 °C for 30 s, 58 °C for 30 s, and 72 °C for 30 s. The primers used included 5′-TCCAGAAGATGGTGCTCTTG-3′ and 5′-CTGGCCCTTCATGTCCTAAA-3′ for the *cgd2_930* gene, 5′-GCTCACTTCCTAACTCCACCAG-3′ and 5′-ACCATCTTGCCACTCTGTTCTT-3′ for the *cgd2_4270* gene, and 5′-CTCCACCAACTAAGAACGGCC-3′ and 5′-TAGAGATTGGAGGTTGTTCCT-3′ for the *18s* rRNA gene [15]. The relative expression levels of *cgd2_930* and *cgd2_4270* genes in various developmental stages were calculated using the delta-delta method [21].

### 2.8. Immunofluorescence Assay (IFA)

For the assessment of CpINS-4 and CpINS-6 expression in developmental stages of *C. parvum*, oocysts and sporozoites were suspended in PBS and dried onto poly-L-lysine-treated microscope slides (Waterborne, New Orleans, LA, USA), whereas HCT-8 cells infected with sporozoites were grown on coverslips for 24 and 48 h, fixed with methanol for 15 min, and permeabilized with 0.5% Triton X-100 for 30 min. After blocking with 5% BSA at room temperature for 1 h, oocysts, sporozoites and infected HCT-8 cells were probed overnight with the polyclonal antibodies (1:500) in 5% BSA/PBS (weight/volume), followed by staining with Alexa Fluor 594-conjugated goat-anti-rabbit IgG (Cell Signaling Technology, Danvers, MA, USA) and nuclear staining with 4′, 6-diamidino-2-phenylindole (DAPI, Sigma-Aldrich). After three washes with PBS, the slides and coverslips were mounted with Antifade Mounting Medium (Booster, Wuhan, China) and examined using differential interference contrast (DIC) and fluorescence microscopy on a BX53 microscope (Olympus, Tokyo, Japan).

### 2.9. Invasion Neutralization Assay

The effect of polyclonal antibodies against CpINS-4 and CpINS-6 on *C. parvum* infection of HCT-8 cells was examined using an in vitro neutralization assay. Hypochlorite-treated oocysts were preincubated at 37 °C in antiserum or preimmune serum diluted with culture medium for 15 min, with the culture medium only as the negative control. They were inoculated into HCT-8 cell culture grown in coverslips in 24-well plates at ~90% confluence as described above. After 2-h incubation, residual oocysts were washed off the monolayers with fresh culture medium. After additional cultivation for 24 h, the monolayers were fixed with methanol and stained with Cy3-labeled Sporo-Glo antibodies (Waterborne, New Orleans, LA, USA). The coverslips were examined by immunofluorescence microscopy. Images of 50 random microscope fields per coverslip were captured under 200× magnification and analyzed using ImageJ (https://imagej.nih.gov/ij/download.html, accessed on 20 December 2020). The percent of invasion inhibition was calculated using the following formula: (1 − [No. of parasites after postimmune serum treatment/No. of parasites after preimmune serum treatment]) × 100%. Three independent experiments were conducted for the determination of percent invasion inhibition by the antibodies alone or in combination.

## 3. Results

### 3.1. Characteristics of INS Encoded by cgd2_930 and cgd2_4270

**Figure 1:**
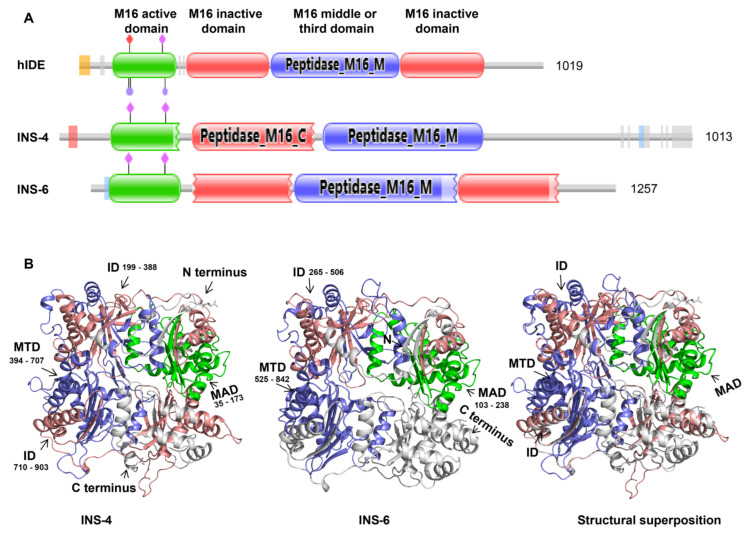
Structure feature of insulinaselike proteases CpINS-4 and CpINS-6 of *Cryptosporidium parvum*. (**A**) Diagram of domains in CpINS-4, CpINS-6, and human insulin degrading enzyme (hIDE). CpINS-4 has all four domains associated with classic insulin degrading enzymes, while CpINS-6 has an M16 active domain, an inactive domain, and one middle domain. (**B**) Predicted tertiary structures of CpINS-4 (left), CpINS-6 (middle) and structural superposition (right). The ribbon model is colored based on the M16 active domain (MAD), inactive domains (ID), and the middle domain (MTD).

The gene encoding CpINS-4, *cgd2_930*, has one exon of 3042 bp without any intron. CpINS-4 has all four domains seen in functional M16 proteases, including a M16 peptidase domain, two M16 inactive domains, and a middle or third domain of peptidase. In contrast, CpINS-6 is a secreted protein with a signal peptide. CpINS-6 is encoded by the 3774-bp *cgd2_4270* gene with no intron. Compared with CpINS-4, the domain of CpINS-6 is incomplete and lacks one of the M16 inactive domains (Figure 1A). The predicted tertiary structures of CpINS-4 and CpINS-6 were modeled based on the crystal structure (PDB ID: 2jbu_B) of human insulin degrading enzyme (hIDE) and the structure of hIDE complex with its copurified peptides [20]. CpINS-4 had 28% identity to PDB ID: 2jbu_B, while CpINS-6 had 19% identity to it. The homology model of PDB ID: 2jbu_B was the best structural template for the construction of three-dimensional structures of *C. parvum* INS. The homology models generated correlated well with the primary sequences of CpINS-4 and CpINS-6. The ribbon model for the predicted structure of CpINS-4 and CpINS-6 consists of antiparallel β-sheets and α-helices, with the C and N termini separating far apart at the bottom of the structure. The N-terminal and C-terminal domains of CpINS-4 and CpINS-6 are connected by a hinge in the third domain. The C-terminal region of CpINS-4 is composed of the part of M16 middle domain and M16 inactive domain, while the C-terminal region of CpINS-6 has the M16 inactive domain missing (Figure 1B). 

### 3.2. Production of Recombinant INS4 and INS6 

**Figure 2:**
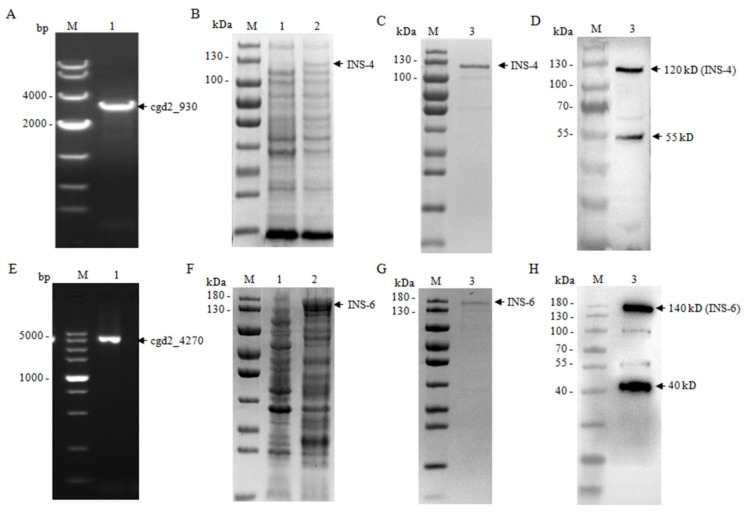
Expression of recombinant CpINS-4 and CpINS-6 in *Escherichia coli.* (**A**–**D**) Expression of CpINS-4 in *E. coli* BL21 (DE3). The *cgd2_930* gene encoding CpINS-4 of *C. parvum* was amplified by PCR; lane M: molecular makers; lane 1: *cgd2_930* PCR product (**A**). The PCR product was cloned and recombinant CpINS-4 was expressed in *E. coli* BL21 (DE3) and analyzed by SDS-PAGE (**B**). The recombinant CpINS-4 was purified, with purity verified by SDS-PAGE and Western blot; lane M: molecular weight makers; lane 1: lysate from recombinant bacteria without IPTG induction; lane 2: lysate from IPTG-induced recombinant bacteria, with the expected product indicated by an arrow; lane 3: CpINS-4 purified using Ni-NAT affinity chromatography (**C**,**D**). (**E**–**H**) Expression of CpINS-6 in *E. coli* BL21 (DE3). The *cgd2_4270* gene encoding CpINS-6 of *C. parvum* was amplified by PCR; lane M: molecular makers; lane 1: *cgd2_4270* PCR product (**E**). The PCR product was cloned and recombinant CpINS-6 was expressed in *E. coli* BL21 (DE3) and analyzed by SDS-PAGE (**F**). The recombinant CpINS-6 was purified, with purity verified by SDS-PAGE and Western blot; lane M: molecular weight makers; lane 1: lysate from recombinant bacteria without IPTG induction; lane 2: lysate from IPTG-induced recombinant bacteria, with the expected product indicated by an arrow; lane 3: CpINS-6 purified using Ni-NAT affinity chromatography (**G**,**H**).

The full *cgd2_930* and *cgd2_4270* genes were amplified by PCR (Figure 2). The PCR products were cloned into the pET-28a vector and expressed in *E. coli* BL21-Star (DE3) pLysS cells (Figure 2). The recombinant CpINS-4 and CpINS-6 proteins generated were purified using histidine-tagged protein purification resins. In SDS-PAGE and Western blot analyses of the recombinant CpINS-4, the size of the recombinant protein was ~120 kDa as expected. However, there were some other bands of ~100 kDa and ~55 kDa that were confirmed to be the fragments of the recombinant protein CpINS-4 using MALDI-TOF-MS analysis. In SDS-PAGE and Western blot analyses of the recombinant CpINS-6, the expected band of ~140 kDa was seen. There were also several smaller bands of ~100 kDa, ~55 kDa, and ~40 kDa of the recombinant INS-6 confirmed by MALDI-TOF-MS analysis.

### 3.3. Identification of Native CpINS-4 and CpINS-6

**Figure 3:**
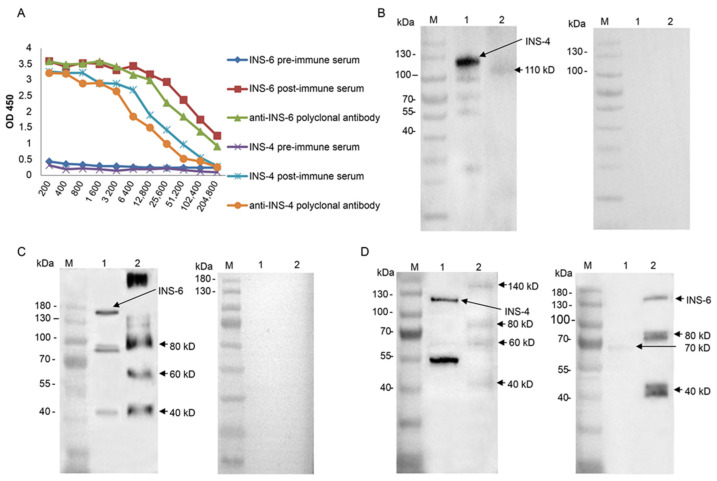
Expression of native CpINS-4 and CpINS-6 and cross-reactivity of polyclonal antibodies among INS proteins. (**A**) Titers of anti-CpINS-4 and anti-CpINS-6 polyclonal antibodies generated by immunizations with recombinant proteins as indicated by ELISA analysis. (**B**) Western blot analysis of native CpINS-4 protein in sporozoite lysate using polyclonal antibodies against the CpINS-4 (left panel) and preimmune sera (right panel); lane M: molecular weight markers; lane 1: purified recombinant CpINS-4 protein; lane 2: crude protein extract of sporozoites. (**C**) Western blot analysis of native CpINS-6 protein in sporozoite lysate using polyclonal antibody against the CpINS-6 (left panel) and preimmune sera (right panel); lane M: molecular weight markers; lane 1: purified recombinant CpINS-6 protein; lane 2: crude protein extract of sporozoites. (**D**) Cross-reactivity of anti-CpINS-4 antibodies (left panel) and anti-CpINS-6 antibodies (right panel) in Western blot analysis of recombinant proteins; lane M: protein size markers; lane 1: purified recombinant CpINS-4; lane 2: purified recombinant CpINS-6.

To characterize native CpINS-4 and CpINS-6, polyclonal antibodies against the recombinant INS were generated in rabbits. In ELISA analysis, the titers of postimmune sera and polyclonal antibodies both reached 1:25,600 (Figure 3A). In Western blot evaluation of CpINS-4, the antibodies against the CpINS-4 recognized the ~120 kDa, ~100 kDa, and ~55 kDa fragments of the recombinant protein and reacted with a protein of ~110 kDa in lysates of *C. parvum* sporozoites (Figure 3B). In Western blot analysis, the antibodies against the CpINS-6 mainly reacted with the recombinant CpINS-6 of ~140 kDa and three smaller fragments. They also recognized the native protein of molecular weight higher than 180 kDa and several smaller products in lysates of *C. parvum* sporozoites (Figure 3C). None of these proteins reacted with preimmune serum, confirming the specificity of the antibodies against CpINS-4 and CpINS-6. In evaluation of the cross-reactivity of the antibodies by Western blot analysis, anti-CpINS-4 antibodies reacted very weakly with fragments of ~140 kDa, ~80 kDa, ~60 kDa and ~40 kDa of recombinant CpINS-6. Similarly, antibodies against the CpINS-6 reacted very weakly with the ~70 kDa fragment of recombinant CpINS-4 (Figure 3D).

### 3.4. Expression of CpINS-4 and CpINS-6 in Life Cycle Stages

**Figure 4:**
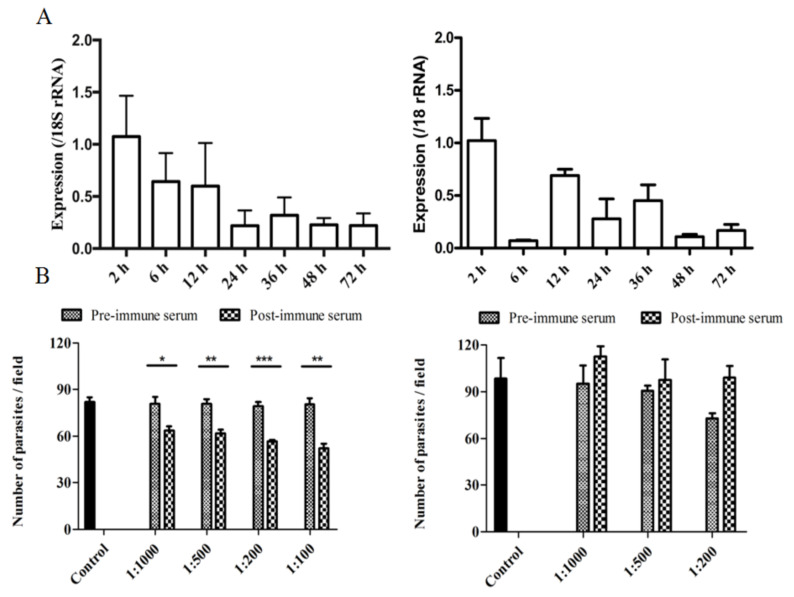
Assessment of biological functions of CpINS-4 and CpINS-6 in *Cryptosporidium parvum.* (**A**) Relative expression level of the *cgd2_930* (left panel) and *cgd2_4270* genes (right panel) at different time points of *C. parvum*-infected HCT-8 culture as determined by qPCR. The error bars presented represent standard deviations of the means from three replicate assays. (**B**) Neutralization efficiency of the polyclonal anti-CpINS-4 (left panel) and anti-CpINS-6 (right panel) antisera on *C. parvum* infection of HCT-8 cells. Hypochlorite-treated oocysts were preincubated with 1:1000, 1:500, 1:200, and 1:100 dilutions of postimmune sera, with preimmune serum as controls. Parasite loads in 50 random 200 × microscope fields were determined and compared with treatment groups. Each bar represents mean ± SD from three replicate assays. * *P* < 0.05, ** *P* < 0.01, *** *P* < 0.001.

To determine the transcriptional profiles of the *cgd2_930* and *cgd2_4270* genes during intracellular development, we assessed the expression of the two genes by qPCR over a 72 h time course of *C. parvum* infection in HCT-8 cells. Both genes had the highest expression at 2 h of the infection. The expression of the *cgd2_930* gene decreased gradually afterwards, while the expression of *cgd2_4270* had additional peaks at 12 h and 36 h (Figure 4A).

**Figure 5:**
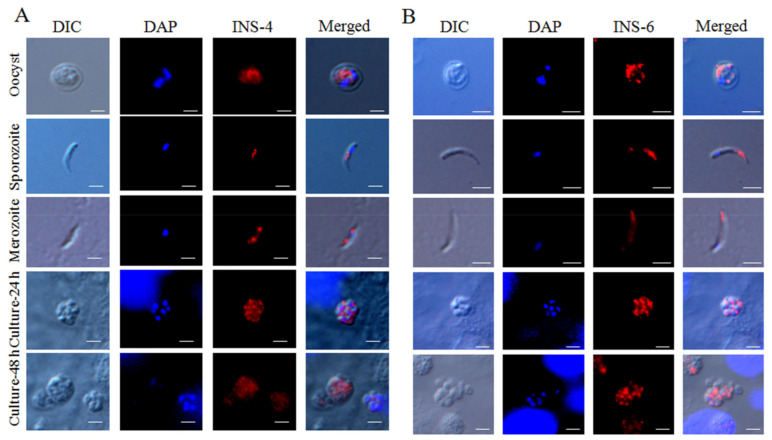
Expression of INS proteins in life cycle stages of *Cryptosporidium parvum*. The expression of CpINS-4 (**A**) and CpINS-6 (**B**) on *C. parvum* oocyst (top panel), sporozoites (second panel), merozoites (third panel) and intracellular developmental stages in HCT-8 cell cultures at 24 h (fourth panel) and 48 h (bottom panel) was assessed by immunofluorescence using the polyclonal antibodies generated. The images were taken under differential interference contrast (DIC), with nuclei being counter-stained with 4′, 6-diamidino-2-phenylindole (DAPI), parasites stained by immunofluorescence assay with anti-INS antibodies, and superimposition of the three images (Merged). Scale-bars: 2 µm for all figures.

The antibodies generated against the CpINS-4 and CpINS-6 were used to examine CpINS-4 and CpINS-6 expression in *C. parvum* sporozoites and intracellular stages by immunofluorescence microscopy. In oocysts, the antibodies against CpINS-4 and CpINS-6 both reacted with sporozoites rather than other oocyst contents. In free sporozoites, the antibodies against CpINS-4 reacted with middle region of sporozoites (Figure 5A, top panel), while at 24 h and 48 h of the in vitro culture, anti-CpINS-4 had the highest reactivity to middle region of merozoites (Figure 5A, middle, fourth and bottom panel). In contrast, polyclonal anti-CpINS-6 antibodies reacted with the apical region of sporozoites and merozoites (Figure 5B).

### 3.5. Inhibition of C. parvum Invasion by CpINS-4 and CpINS-6 Antibodies

The neutralizing effect of postimmune sera against CpINS-4 and CpINS-6 on *C. parvum* invasion was assessed in vitro. In comparison with the preimmune sera-treated culture, the mean parasite load was reduced significantly when HCT-8 cell cultures were infected with sporozoites treated with postimmune sera against CpINS-4. The mean numbers of parasites were 63.7 ± 2.7, 61.8 ± 2.3, 56.5 ± 1.0, and 52.0 ± 3.0 per 200 × field in *C. parvum* cultures treated with postimmune sera against CpINS-4 diluted 1:1000, 1:500, 1:200, and 1:100, respectively, compared with 80.8 ± 4.4, 80.8 ± 3.0, 79.4 ± 2.4, and 80.4 ± 4.1 in cultures treated preimmune sera diluted 1:1000, 1:500, 1:200, and 1:100, respectively. Therefore, the antisera inhibited *C. parvum* infection by 21.3% (t_(2)_ = 3.31, *P* = 0.0296), 23.7% (t_(2)_ = 5.03, *P* = 0.0074), 29% (t_(2)_ = 8.80, *P* = 0.0001), and 36.0% (t_(2)_ = 5.59, *P* = 0.005) at 1:1000, 1:500, 1:200, and 1:100 dilutions, respectively. In contrast, antisera against CpINS-6 did not have significant effects on *C. parvum* invasion. The mean numbers of parasites were 95.2 ± 11.6 and 112.6 ± 6.5 (t_(2)_ =1.311, P = 0.26) per 200 × field for pre and postimmune sera at the dilution of 1:1000, respectively, 90.6 ± 3.5 and 97.5 ± 13.1(t_(2)_ = 0.508, P = 0.638) per 200 × field for pre and postimmune sera at the dilution of 1:500, respectively, and 72.8 ± 3.2 and 99.2 ± 7.1 (t_(2)_ = 3.362, *P* = 0.0283) per 200 × field for pre and postimmune sera at the dilution of 1:200, respectively (Figure 4B).

## 4. Discussion

The data from the present study suggest that CpINS-4 and CpINS-6 have very different structures and are expressed in different organelles of *C. parvum*, thus may play different roles in the invasion and growth of the pathogen. CpINS-4 has all four conserved domains in functional M16 proteases, while a peptidase M16 inactive domain is absent in CpINS-6. Furthermore, there is only 15.2% identity in amino acids between CpINS-4 and CpINS-6. The differences in functional domains and amino acid sequences have probably led to completely different tertiary structures of the two INS. Like other M16A proteases subfamily, CpINS-4 and CpINS-6 both adopt a clamshell structure, with the N- and C-terminal domains forming a hydrolytically active central cavity (Figure 1B). The opening and closing of the N- and C-terminal domains are required to facilitate the access of the substrate to the hydrolytically active site and represent a rate-limiting step within the INS catalytic mechanism [10], while the absence of a peptidase M16 inactive domain in C-terminal domains of CpINS-6 may directly affect the function of the C-terminal domains and clamshell structure switch, thereby changing the catalytic activity on the substrate.

The recombinant CpINS-4 and CpINS-6 appear to be processed proteolytically. Multiple products were observed in *E. coli*-expressed recombinant INS proteins and native CpINS-4 and CpINS-6. It is possible that both CpINS-4 and CpINS-6 sequences contain proteolytic cleavage sites. In sequence analysis, CpINS-4 has eight putative SΦX(E/D) cleavage sites with the sequences SPED (265–268), SFSD (543–546), SALD (973–976), SIME (261–264), SVIE (361–364), SLDE (401–404), SIPE (454–457), and SFFE (659–662), which in theory can lead to the hydrolysis of the full-CpINS-4 into several fragments. Similarly, CpINS-6 has four putative SΦX (E/D) cleavage sites, with the sequences SIWD (23–26), SVSE (197–200), SIFE (334–337), SVSD (401–404). These cleavage sites may be responsible for the proteolytic processing of CpINS-4 and CpINS-6 in a way similar to the post-translational processing of rhoptry proteins in *T. gondii*. In *T. gondii*, the N-terminal prodomain of ROP1 is processed at its SΦXE cleavage site [22], while the proteolytical processing of TLN1 occurs at the cleavage site SΦXD [13]. TLN1 is a rhoptry metalloprotease and targeted to the rhoptry via an N-terminal prodomain, which is processed at SΦX (E/D) cleavage sites. After proteolytic processing and removing the organelle-targeting sequences in toxolysins, the C-terminal domain is associated tightly with the N-terminal domain, forming homo-oligomers. The proteolytic processing may be an evolutionary adaptation in order to alter the substrate of TLN1 [13]. Two other rhoptry proteins that are involved in virulence of *T. gondii*, ROP17 and ROP18, are also predicted to have several SΦX (E/D) cleavage sites [23,24].

CpINS-6 may be present in a high molecular weight complex in *C. parvum*. In the analysis of native CpINS-4 and CpINS-6, the polyclonal antibodies against CpINS-6 reacted with a protein with a higher molecular weight than 180 kDa in lysates of *C. parvum*. As a secreted protein, CpINS-6 could initially exist as a large precursor, which is processed into multiple fragments within the secretory system of the parasite. In *T. gondii*, TLN4 is initially synthesized as a large (~260 kDa) precursor, and subsequently processed into multiple fragments that remain associated with each other in a large molecular complex [12]. The large protein could also be a mixed dimer. Insulin proteases are known to be active as a homodimer containing one active subunit and another subunit that binds substrate with normal affinity [25]. In addition, the C-terminal domain is also required for dimerization [9], suggesting that C-terminal domain might play an important role in the formation of a high molecular weight complex of CpINS-6.

CpINS-4 and CpINS-6 may have classic INS activities. In the INS of *C. parvum*, CpINS-4 has four conserved domains, with the N-terminal domain containing the inverted Zn^2+^-binding motif HFLEH in amino acids 69–73. Although CpINS-6 may be not a classic insulinlike protease with all four conserved domains, its N-terminal domain does contain the inverted Zn^2+^-binding motif HLMEH in amino acids 126–130. The inverted Zn2+-binding motif is a key feature of M16 proteases, is required for catalytic activity, and affects substrate binding. Further studies should be conducted to determine whether CpINS-4 and CpINS-6 both have proteolytic activities associated with classic insulinaselike protease. 

CpINS-4 could be involved in the invasion process of *C. parvum*. Various proteases and protein kinases are secreted by the secretory organelles of apicomplexans parasites to counteract the host immune responses [6]. In agreement with its potential role in the invasion of host cells, CpINS-4 is expressed in both sporozoites and merozoites, the invasion stages of the parasites, mainly in the middle region. Previously, based on sequence homology, CpINS-4 was indicated as a putative rhoptry protein [26]. While the protein appears to be processed in the same way as some rhoptry proteins in *T. gondii*, its expression pattern in both sporozoites and merozoites revealed by immunofluorescence is more in line with a location in dense granules. The early expression level of the gene encoding it during the in vitro culture and the partial neutralization effect of antibodies against it support the role of CpINS-4 in host cell invasion. Compared with CpINS-4, the location of CpINS-6 expression appears different; its expression in both spozoites and merozoites is confined to apical end, more in line with a rhoptry or microneme location. While the *cgd2_4270* gene encoding CpINS-6 was the highest at 2 h of in vitro culture, antibodies against it failed in neutralizing the invasion of host cells by sporozoites.

In conclusion, the preliminary study represents our initial attempt in characterizing the functions of CpINS-4 and CpINS-6 in *C. parvum*. Although the two INS both have the active Zn-chelating “HXXEH” motif and similar post-transcriptional processing, they appear to be very different in structure, are expressed in different organelles, and play different roles in the invasion and growth of the parasites. Further studies using more advanced tools are needed to understand their precise locations and functions in *Cryptosporidium* spp.

## Data Availability

Not applicable.

## Abbreviations

The following abbreviations are used in this manuscript:

INS	Insulin-like protease
CpINS-4	*Cryptosporidium parvum* INS-4
CpINS-6	*Cryptosporidium parvum* INS-6
Intron	Sequences that block linear gene expression
MALDI-TOF-MS	Matrix Assisted Laser Desorption Ionization-Time of Flight-Mass spectrometry
SDS-PAGE	Sodium salt-polyacrylamide gel electrophoresis
PVDF	Polyvinylidene Fluoride
ELISA	Enzyme Linked Immune Sorbent Assay
BSA	Bovine Serum albumin
PBS	Phosphate Buffered Saline
FBS	Fatal Bovine Serun
ECL	Electro-Chemi-Luminescence
RIPA	Radio-Immunoprecipitation Assay
qPCR	Real-time Quantitative Polymerase Chain Reaction

## References

[1] Feng Y., Ryan U.M., Xiao L. (2018). Genetic diversity and population structure of *Cryptosporidium*. Trends Parasitol..

[2] Checkley W., White A.C., Jaganath D., Arrowood M.J., Chalmers R.M., Chen X.M., Fayer R., Griffiths J.K., Guerrant R.L., Hedstrom L. (2015). A review of the global burden, novel diagnostics, therapeutics, and vaccine targets for *Cryptosporidium*. Lancet Infect Dis..

[3] Kotloff K.L., Nataro J.P., Blackwelder W.C., Nasrin D., Farag T.H., Panchalingam S., Wu Y., Sow S.O., Sur D., Breiman R.F. (2013). Burden and aetiology of diarrhoeal disease in infants and young children in developing countries (the global enteric multicenter study, gems): A prospective, case-control study. Lancet.

[4] Khalil I.A., Troeger C., Rao P.C., Blacker B.F., Brown A., Brewer T.G., Colombara D.V., De Hostos E.L., Engmann C., Guerrant R.L. (2018). Morbidity, mortality, and long-term consequences associated with diarrhoea from *Cryptosporidium* infection in children younger than 5 years: A meta-analyses study. Lancet Glob. Health.

[5] Chavez M.A., White A.C. (2018). Novel treatment strategies and drugs in development for cryptosporidiosis. Expert Rev. Anti Infect. Ther..

[6] Hunter C.A., Sibley L.D. (2012). Modulation of innate immunity by *Toxoplasma gondii* virulence effectors. Nat. Rev. Microbiol..

[7] Becker A.B., Roth R.A. (1992). An unusual active site identified in a family of zinc metalloendopeptidases. Proc. Natl. Acad. Sci. USA.

[8] Taylor A.B., Smith B.S., Kitada S., Kojima K., Miyaura H., Otwinowski Z., Ito A., Deisenhofer J. (2001). Crystal structures of mitochondrial processing peptidase reveal the mode for specific cleavage of import signal sequences. Structure.

[9] Li P., Kuo W.L., Yousef M., Rosner M.R., Tang W.J. (2006). The c-terminal domain of human insulin degrading enzyme is required for dimerization and substrate recognition. Biochem. Biophys. Res. Commun..

[10] Shen Y., Joachimiak A., Rosner M.R., Tang W.J. (2006). Structures of human insulin-degrading enzyme reveal a new substrate recognition mechanism. Nature.

[11] Murata C.E., Goldberg D.E. (2003). Plasmodium falciparum falcilysin: A metalloprotease with dual specificity. J. Biol. Chem..

[12] Laliberte J., Carruthers V.B. (2011). *Toxoplasma gondii* toxolysin 4 is an extensively processed putative metalloproteinase secreted from micronemes. Mol. Biochem. Parasitol..

[13] Hajagos B.E., Turetzky J.M., Peng E.D., Cheng S.J., Ryan C.M., Souda P., Whitelegge J.P., Lebrun M., Dubremetz J.F., Bradley P.J. (2012). Molecular dissection of novel trafficking and processing of the *Toxoplasma gondii* rhoptry metalloprotease toxolysin-1. Traffic.

[14] Liu S., Roellig D.M., Guo Y., Li N., Frace M.A., Tang K., Zhang L., Feng Y., Xiao L. (2016). Evolution of mitosome metabolism and invasion-related proteins in *Cryptosporidium*. BMC Genom..

[15] Mauzy M.J., Enomoto S., Lancto C.A., Abrahamsen M.S., Rutherford M.S. (2012). The *Cryptosporidium parvum* transcriptome during in vitro development. PLoS ONE.

[16] Zhang S., Wang Y., Wu H., Li N., Jiang J., Guo Y., Feng Y., Xiao L. (2019). Characterization of a species-specific insulinase-like protease in *Cryptosporidium parvum*. Front. Microbiol..

[17] Xu R., Guo Y., Li N., Zhang Q., Wu H., Ryan U., Feng Y., Xiao L. (2019). Characterization of ins-15, a metalloprotease potentially involved in the invasion of *Cryptosporidium parvum*. Microorganisms.

[18] Ni N., Jia R., Guo Y., Li N., Wu H., Feng Y., Xiao L. (2020). Expression and functional studies of ins-5, an insulinase-like protein in *Cryptosporidium parvum*. Front. Microbiol..

[19] Cevallos A.M., Bhat N., Verdon R., Hamer D.H., Stein B., Tzipori S., Pereira M.E., Keusch G.T., Ward H.D. (2000). Mediation of *Cryptosporidium parvum* infection in vitro by mucin-like glycoproteins defined by a neutralizing monoclonal antibody. Infect. Immun..

[20] Im H., Manolopoulou M., Malito E., Shen Y., Zhao J., Neant-Fery M., Sun C.Y., Meredith S.C., Sisodia S.S., Leissring M.A. (2007). Structure of substrate-free human insulin-degrading enzyme (ide) and biophysical analysis of atp-induced conformational switch of ide. J. Biol. Chem..

[21] Livak K.J., Schmittgen T.D. (2001). Analysis of relative gene expression data using real-time quantitative pcr and the 2(-delta delta c(t)) method. Methods.

[22] Bradley P.J., Hsieh C.L., Boothroyd J.C. (2002). Unprocessed *Toxoplasma* rop1 is effectively targeted and secreted into the nascent parasitophorous vacuole. Mol. Biochem. Parasitol..

[23] El Hajj H., Lebrun M., Arold S.T., Vial H., Labesse G., Dubremetz J.F. (2007). Rop18 is a rhoptry kinase controlling the intracellular proliferation of *Toxoplasma gondii*. PLoS Pathog..

[24] Hajj H.E., Lebrun M., Fourmaux M.N., Vial H., Dubremetz J.F. (2006). Characterization, biosynthesis and fate of rop7, a rop2 related rhoptry protein of *Toxoplasma gondii*. Mol. Biochem. Parasitol..

[25] Song E.S., Juliano M.A., Juliano L., Hersh L.B. (2003). Substrate activation of insulin-degrading enzyme (insulysin). A potential target for drug development. J. Biol. Chem..

[26] Sanderson S.J., Xia D., Prieto H., Yates J., Heiges M., Kissinger J.C., Bromley E., Lal K., Sinden R.E., Tomley F. (2008). Determining the protein repertoire of *Cryptosporidium parvum* sporozoites. Proteomics.

